# Orally administrated *Lactobacillus gasseri* TM13 and *Lactobacillus crispatus* LG55 can restore the vaginal health of patients recovering from bacterial vaginosis

**DOI:** 10.3389/fimmu.2023.1125239

**Published:** 2023-07-27

**Authors:** Fengyuan Qi, Shangrong Fan, Chao Fang, Lan Ge, Jinli Lyu, Zhuoqi Huang, Shaowei Zhao, Yuanqiang Zou, Liting Huang, Xinyang Liu, Yiheng Liang, Yongke Zhang, Yiyi Zhong, Haifeng Zhang, Liang Xiao, Xiaowei Zhang

**Affiliations:** ^1^ Department of Obstetrics and Gynecology, Peking University Shenzhen Hospital, Shenzhen, China; ^2^ BGI-Shenzhen, Shenzhen, China; ^3^ ShenZhen Engineering Laboratory of Detection and Intervention of Human Intestinal Microbiome, Shenzhen, China; ^4^ College of Life Sciences, University of Chinese Academy of Sciences, Beijing, China; ^5^ Institute of Obstetrics and Gynecology, Shenzhen Peking University Hong Kong University of Science and Technology Medical Center, Shenzhen, China; ^6^ Shenzhen Key Laboratory on Technology for Early Diagnosis of Major Gynecological Diseases, Peking University Shenzhen Hospital, Shenzhen, China; ^7^ BGI Precision Nutrition (Shenzhen) Technology Co., Ltd, Shenzhen, China; ^8^ Department of Biology, School of Life Sciences, Southern University of Science and Technology, Shenzhen, China

**Keywords:** bacterial vaginosis, *Lactobacillus*, oral administration, vaginal microbiome, *Prevotella timonensis*

## Abstract

Bacterial vaginosis (BV) is a common infection of the lower genital tract with a vaginal microbiome dysbiosis caused by decreasing of lactobacilli. Previous studies suggested that supplementation with live *Lactobacillus* may benefit the recovery of BV, however, the outcomes vary in people from different regions. Herein, we aim to evaluate the effectiveness of oral Chinese-origin *Lactobacillus* with adjuvant metronidazole (MET) on treating Chinese BV patients. In total, 67 Chinese women with BV were enrolled in this parallel controlled trial and randomly assigned to two study groups: a control group treated with MET vaginal suppositories for 7 days and a probiotic group treated with oral *Lactobacillus gasseri* TM13 and *Lactobacillus crispatus* LG55 as an adjuvant to MET for 30 days. By comparing the participants with Nugent Scores ≥ 7 and < 7 on days 14, 30, and 90, we found that oral administration of probiotics did not improve BV cure rates (72.73% and 84.00% at day 14, 57.14% and 60.00% at day 30, 32.14% and 48.39% at day 90 for probiotic and control group respectively). However, the probiotics were effective in restoring vaginal health after cure by showing higher proportion of participants with Nugent Scores < 4 in the probiotic group compared to the control group (87.50% and 71.43% on day 14, 93.75% and 88.89% on day 30, and 77.78% and 66.67% on day 90). The relative abundance of the probiotic strains was significantly increased in the intestinal microbiome of the probiotic group compared to the control group at day 14, but no significance was detected after 30 and 90 days. Also, the probiotics were not detected in vaginal microbiome, suggesting that *L. gasseri* TM13 and *L. crispatus* LG55 mainly acted through the intestine. A higher abundance of *Prevotella timonensis* at baseline was significantly associated with long-term cure failure of BV and greatly contributed to the enrichment of the lipid IVA synthesis pathway, which could aggravate inflammation response. To sum up, *L. gasseri* TM13 and *L. crispatus* LG55 can restore the vaginal health of patients recovering from BV, and individualized intervention mode should be developed to restore the vaginal health of patients recovering from BV.

**Clinical trial registration:**

https://classic.clinicaltrials.gov/ct2/show/, identifier NCT04771728.

## 1 Introduction

Bacterial vaginosis (BV) is a microecological disorder caused by decreased abundance of lactobacilli and an increased abundance of anaerobic bacteria, commonly affecting the female lower genital tract (1). The prevalence of BV is around 20%-30% all over the world (2). BV increases the susceptibility of women of reproductive age to sexually transmitted infections (STIs), including human immunodeficiency virus (HIV) (3), human papillomavirus (HPV) (4), and gonorrhea (5), and increases the risk of spontaneous abortion, preterm delivery, and amniotic fluid infection during pregnancy (6–9). Common symptoms of BV include elevated vaginal pH, increased leukorrhea, odor, vulvar itching, and burning pain. In addition, approximately 50% of patients are asymptomatic (3, 10, 11).

16S rRNA amplicon sequencing technology has been widely used to characterize the microbiome of the vagina in healthy or BV states (12–14). In BV patients, *Lactobacillus* spp. are replaced by anaerobic bacteria such as *Gardnerella* spp., *Prevotella* spp., *Mobiluncus* spp., and *Atopobium vaginae*, which result in high production of cadaveric amines, putrescine, succinate, and acetate leading to the altered chemical composition of secretions as well as higher pH (15). However, 16S rRNA amplicon sequencing has low species resolution and functional information of microorganisms cannot be directly obtained from sequence data. Since metagenomic shotgun sequencing is able to identify microbial categories with high resolution and characterize their biological functions (16, 17), it has been wildly used in explaining the structure and function of the vaginal microbiome (18, 19). It’s no doubt that metagenomic shotgun sequencing is a better tool to study the mechanisms of vaginal microbiome involvement in pathogenesis of BV.

Metronidazole (MET) has been recommended by the Centers for Disease Control and Prevention as a treatment method for BV (20). Yet, the 12-month long-term cure rate is only 30% (21). The inhibition of MET penetration by biofilms is thought to be the main cause of BV recurrence (22). Probiotics have been used as an alternative treatment approach to prevent recurrent BV. For example, previous studies have reported that oral administration of *Lactobacillus* as an adjuvant therapy could increase the long-term cure rate of BV by improving the balance of the vaginal microbiome (23–26). Microbial translocation from the colon to the vagina has been hypothesized as a potential pathway for the efficacy of oral probiotics (27, 28). Orally administrated probiotics could also suppress systemic inflammatory responses via the fermentation products of probiotics in the intestine (29). However, the outcome of the probiotic treatment on BV could vary in different ethnic groups since the vaginal microbiome is different among women in different ethnic groups (30). A study demonstrated that oral *L. rhamnosus* GR-1 and *L. reuteri* RC-14, isolated from European women, were barely detectable in the intestine and vaginal microbiome after administration (31). It has also been confirmed that native dominant *Lactobacillus* could persistently colonize in the vagina compared to others (32). This suggests that isolated probiotic strains are more likely to function in women of the same ethnic groups, but more evidences should be provided.

The probiotic strains used in this study are *L. gasseri* TM13 and *L. crispatus* LG55 which were isolated from the feces of 2 healthy Chinese people, which we developed in-house. They showed the strong ability to lower vaginal pH, inhibit the growth of pathogenic bacteria and fungi, and alleviated the inflammatory response of BV rats (33). In this study, we aim at evaluating the effectiveness of oral Chinese-origin probiotic strains, *L. gasseri* TM13 and *L. crispatus* LG55, with adjuvant MET in treating Chinese BV patients, and investigate the dynamic of the intestine and vaginal microbiome using metagenomic sequencing during the trial.

## 2 Materials and methods

### 2.1 Trial population

Women who attended the gynecology outpatient clinic of Peking University Shenzhen Hospital in China between June 2020 and April 2021 and presented with abnormal leucorrhoea symptoms were enrolled in this single-center, prospective, parallel-group, randomized controlled clinical trial. Inclusion criteria were: age of 18 - 55 years, premenopause, with a history of sexual activity, and a Nugent Score ≥ 7. Exclusion criteria were: vulvovaginal candidiasis (VVC), trichomonas vaginalis (TV) infection, *Chlamydia trachomatis* (CT) infection, gonococcal vaginitis, pregnant or planning to become pregnant, breastfeeding, pelvic inflammatory disease, allergic to MET, on antibiotic therapy, long-term contraceptive or immunosuppressive drug use or allergic, no regular sexual partner (RSP), and those with a history of systemic organic disease or psychiatric disorders.

This study was approved by the Peking University Shenzhen Hospital Medical Ethics Committee (ID: PUshenzhenH2020-009) and published on ClinicalTrials.gov (NCT04771728). Written consent was obtained from all subjects for this study.

### 2.2 Study design

Patients with an initial Nugent Score ≥ 7 were informed of the study protocol. Those who met the criteria signed an informed consent form before the trial. Subjects were asked to complete a questionnaire containing information on demographic characteristics, vaginal health status, history of drug allergies, and history of reproductive system disorders. A random number table generated by SPSS 13.0 software was used to randomly assign subjects to the control or probiotic group using a 1:1 ratio. After enrollment, the probiotic group received MET vaginal suppositories (200 mg daily for 7 days) along with oral intake of probiotic solid drinks containing *L. gasseri* TM13 and *L. crispatus* LG55 (33) (daily intake ≥ 5×10^9^ CFU for 30 days); the control group received the same dose of MET vaginal suppositories.

**Figure 1:**
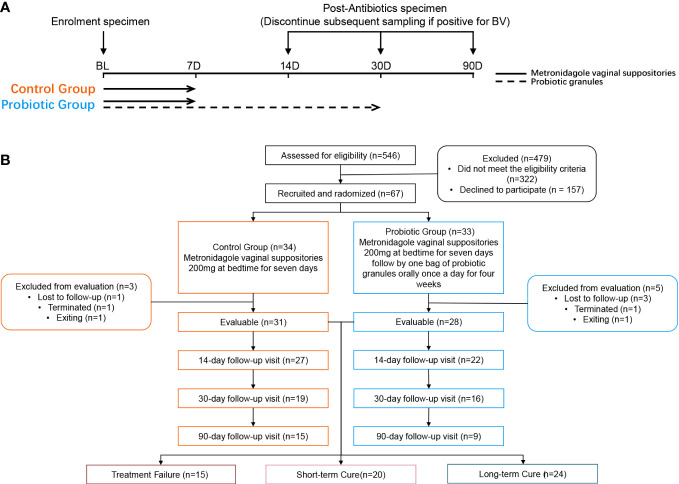
Study design **(A)** and participant flowchart **(B)**.

Follow-up visits were performed on day 14, 30, and 90 after the initiation of treatment. The investigator at enrolment distributed the intervention product. At each follow-up visit, the subject’s compliance, frequency of sex, vaginal health, and the occurrence of adverse events (AEs) were assessed by questionnaire. Subjects were required to avoid topical vaginal dosing during menstruation but continue to take the intervention product orally (**Figure 1A**). In addition, subjects were required to avoid sexual intercourse and vaginal douching throughout the follow-up period. We regrouped all subjects according to treatment outcomes. Subjects who were not found to be BV-negative at any of the follow-up visits were assigned to the treatment failure group; subjects who changed from BV-negative to BV-positive at any of the follow-up visits were assigned to the short-term cure group, and subjects who tested BV-negative at all follow-up visits were assigned to the long-term cure group.

### 2.3 Efficacy evaluation

Before treatment, all subjects were assessed for between-group differences in demographic characteristics, including age, height, weight, marital status, history of smoking, drug allergies, history of childbirth, and history of reproductive disorders. The diagnostic criteria for BV were based on the Nugent Score (34) of vaginal smears. Gram-stained smears were examined under a microscope, and different morphological bacterial cells were counted at 1000× magnification: 0-6 points were diagnosed as BV-negative and 7-10 points as BV-positive. Two cytology technicians performed Gram staining for a double-blind analysis. Clinical testing was also performed for pH, Donders LBG (35), and vaginal cleanliness grades. Efficacy outcomes were assessed by the percentage of subjects with no diagnosis of BV (diagnostic criteria of Nugent Score ≤ 7) at each follow-up visit as the cure rate, based on compliance with protocol set (PP) analysis. Those with a Nugent Score ≥7 were excluded from the clinical trial at each follow-up visit, but these subjects were still included when we assessed cure rates at subsequent follow-up time-points.

### 2.4 Metagenomic sequencing and biological information annotation

Fecal and vaginal samples collected from participants during the initial and follow-up consultations were subjected to DNA extraction and metagenomic shotgun sequencing. Metagenomic shotgun sequencing (100bp paired reads) was performed on the DNBSEQ™ platform (25, 36, 37). Sequencing data were quality-controlled using fastp v0.20.1 (http://github.com/OpenGene/fastp) (38), filtering low-quality sequences with default parameters. High-quality sequences were then aligned to the hg38 human reference gene set using Bowtie2 v2.4.2 (http://github.com/BenLangmead/bowtie2) (39) to exclude the host genome. The de-hosted high-quality sequences were aligned using MetaPhlAn v3.0.7 (http://huttenhower.sph.harvard.edu/metaphlan) as well as HUMAnN 3.0 (http://huttenhower.sph.harvard.edu/humann3) for taxonomic as well as functional annotation (40).

### 2.5 Statistical analysis

The Shannon and Simpson index and Bray-Curtis distance were used to calculate alpha diversity and beta diversity based on the relative abundance of species. The t-test and Mann-Whitney U-test were used to test for differences between two numerical variables in two groups, with 0.05 considered as the threshold for significant differences in p-values. The Chi-square test and Fisher’s exact test were used for differences between groups for categorical variables. Ward Linkage hierarchical clustering of species-level relative abundance of vaginal microorganisms using R Stats v3.5.3 with Jensen-Shannon distances. LEfSe (41) was used to identify species that differed between different treatment outcomes, and a general linear model by MaAsLin2 (42) was used to look for the significantly different pathways. In order to construct co-occurrence networks, we first screened for species with mean relative abundance ≥ 0.1%, then calculated interspecific Spearman correlation coefficients, and constructed co-occurrence networks for two different treatment outcome groups using all correlations with p-values ≤ 0.05, which were visualized by Gephi.

## 3 Results

### 3.1 Clinical trial process and efficacy evaluation

After screening 546 BV patients with Nugent Score ≥7 according to the inclusion and exclusion criteria, 67 patients were included in the clinical trial and randomized. Among them, 8 subjects were excluded (4 subjects were lost to follow-up, 2 subjects were terminated due to non-medication as prescribed, and 2 subjects withdrew from this study for personal reasons). Ultimately, 31 subjects in the control group and 28 subjects in the probiotic group were included in the efficacy evaluation and microbiome analysis (**Figure 1B**). Subjects’ demographic and pathological characteristics at baseline did not differ significantly between the probiotic and control groups (all P > 0.05) (**Table 1**).

**Table 1:** Demographic and behavioral characteristics of subjects at baseline.

	Probiotic group(n=28)	Control group(n=31)	*P*
Individual characteristic			
Age (years, mean ± SD)	34.3 ± 7.7	32.1 ± 6.8	0.249
Height (cm, mean ± SD)	160.9 ± 5.8	161 ± 4.9	0.943
Weight (kg, mean ± SD)	54.7 ± 14.0	51.7 ± 8.6	0.320
Smoking history, n (%)	1 (3.57)	3 (9.68)	0.614
Current relationship status, n (%)			
Married	19 (67.86)	16 (51.61)	0.289
Single, divorced or others	9 (32.14)	15 (48.39)	
Contraception, n (%)	21 (75.00)	15 (48.39)	0.060
Pregnancies num (n, mean ± SD)	2.2 ± 1.8	1.6 ± 1.7	0.130
Delivery num (n, mean ± SD)	1.1 ± 1.0	0.8 ± 1.1	0.279
Clinical symptoms or signs, n (%)			
Abnormal vaginal discharge	26 (92.85)	31 (100)	0.221
Abnormal vaginal odor	27 (96.43)	31 (100)	0.475
Itching	8 (28.57)	7 (22.58)	0.564
Burning	1 (3.57)	1 (3.23)	1.000
Laboratory test			
Cleaning degree, n (%)			
3	27 (96.4)	29 (93.5)	1.000
4	1 (3.57)	2 (6.45)	
Nugent Score (mean ± SD)	8.04 ± 0.64	8.03 ± 0.71	0.955
Donders, n (%)			
I	1 (3.57)	0 (0)	0.567
II b	1 (3.57)	1 (3.23)	
III	26 (92.85)	30 (96.77)	
Vaginal pH (mean ± SD)	4.78 ± 0.16	4.86 ± 0.22	0.119

**Figure 2:**
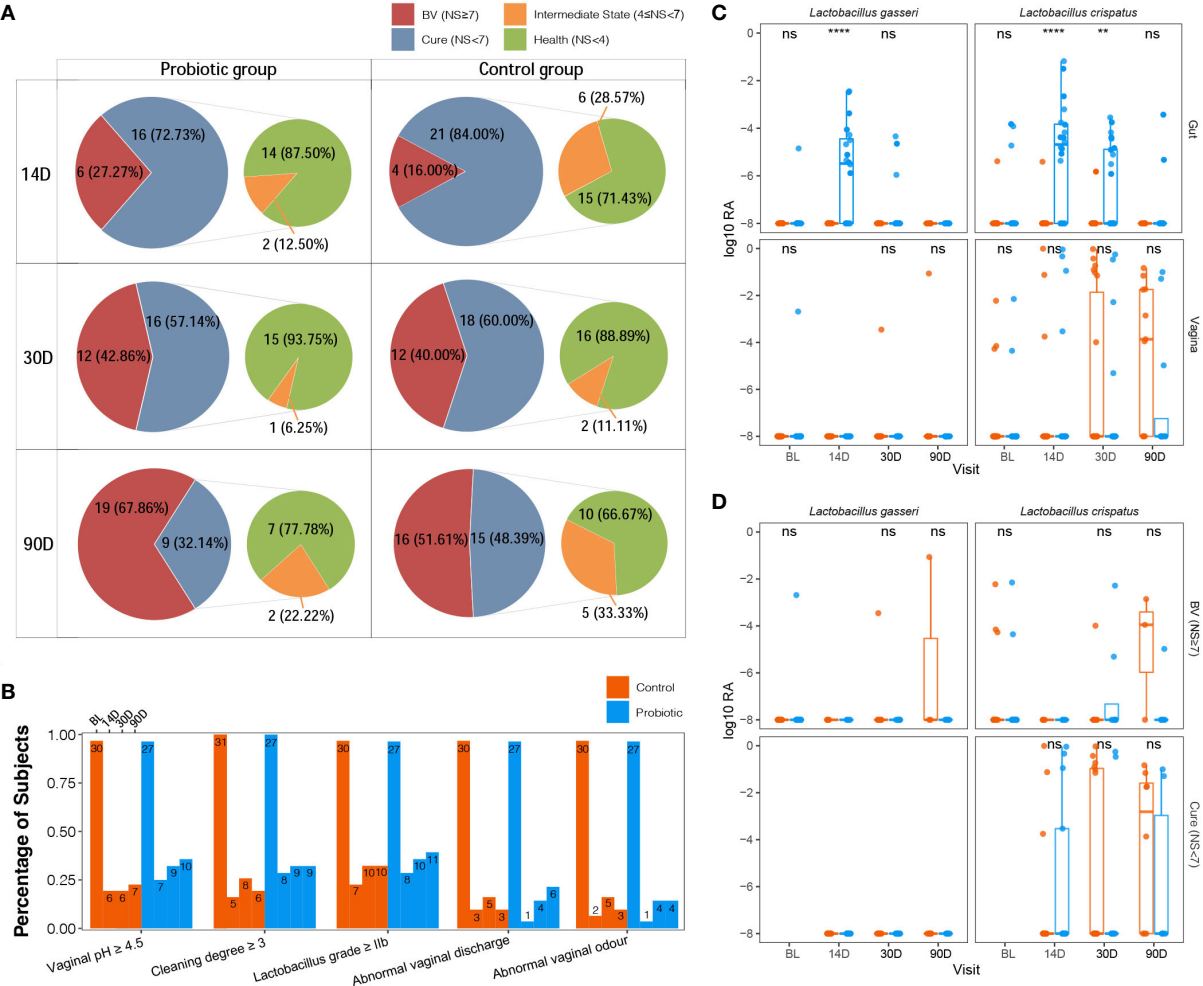
Although orally administrated *L. gasseri* TM13 and *L. crispatus* LG55 preparation cannot improve BV cure rates, it restores the vaginal health after cure mainly acting through the intestine. The number and percentage of the participants with different disease states (BV, non-BV, health, and transition stage) at different time-points of the trial were shown in pie charts **(A)**. The percentage of the participants with detected BV-associated symptoms (abnormal vaginal discharge and abnormal vaginal odor) and laboratory parameters (vaginal pH, vaginal cleaning degree and Donders *Lactobacillus* grade) at different time-points of the trial were shown as a histogram **(B)**. The number marked on the top of the bar denote the case number. Box-and-whisker plots showing the relative abundance of intervention species between groups at different time-points in the intestine or vagina **(C)**. All vaginal samples were grouped according to the Nugent Scores ≥ 7 (BV) and < 7 (cure) **(D)**. Mann-Whitney U test were used to perform the statistical analysis. ** stands for P < 0.01; **** stands for P < 0.0001. ns stands for no significant different (P ≥ 0.05). NS is short for Nugent Score.

At all time-points, there was no significant improvement in the cure rate in the probiotic group compared to the control group. The cure rates for the probiotic and control groups were 72.73% and 84.00% at day 14, respectively; 57.14%, and 60.00% at day 30, respectively; 32.14% and 48.39% at day 90, respectively. Notably, in the group of cured participants, the proportion of those fully recovered (Nugent Score < 4) was higher in the probiotic group compared to the control group (87.50% and 71.43% on day 14, 93.75% and 88.89% on day 30, and 77.78% and 66.67% on day 90, respectively) (**Figure 2A**). To sum up, this suggests that oral administration of *L. gasseri* TM13 with *L. crispatus* LG55 cannot improve BV cure rates but has a role in restoring vaginal health after cure. We also detected BV-associated symptoms and laboratory parameters at different time-points of the trial, however, no significant inter-group differences were found (**Figure 2B**).

### 3.2 Inter-group differences in the abundance and microbial diversity of the intervention strains

In the fecal samples, the relative abundance of *L. crispatus* and *L. gasseri* was significantly higher in the probiotic group than in the control group on day 14 (P < 0.001). On day 30, only *L. crispatus* remained significantly different (P = 0.0037), and the relative abundance of both species in the probiotic group decreased compared to day 14. On day 90, the presence of the *L. gasseri* was not observed in the intestine, and the presence of the *L. crispatus* was detected in only 2 participants (**Figure 2C**). This indicated that the colonization ability of *L. gasseri* TM13 in intestine was not as strong as *L. crispatus* LG55. Furthermore, there was no difference in the relative abundance of *L. crispatus* and *L. gasseri* in the vaginal microbiome between probiotic and control groups (all P > 0.05) (**Figure 2C**), as well as between BV and cure groups (**Figure 2D**), which suggested that no transfer of the intervention strains from the intestine to the vagina was detected.

In addition, to investigate the effect of oral probiotics on the microbial community structure of the intestine and vagina, we observed the differences in alpha-diversity and beta-diversity in the intestine and vaginal microbiome before and after the intervention. No difference in microbial diversity was found between the probiotic and control groups, either in the intestine or the vaginal microbiome (all P > 0.05) (**Figure S1**).

### 3.3 Heterogeneity of the vaginal microbiome and association with treatment outcomes

**Figure 3:**
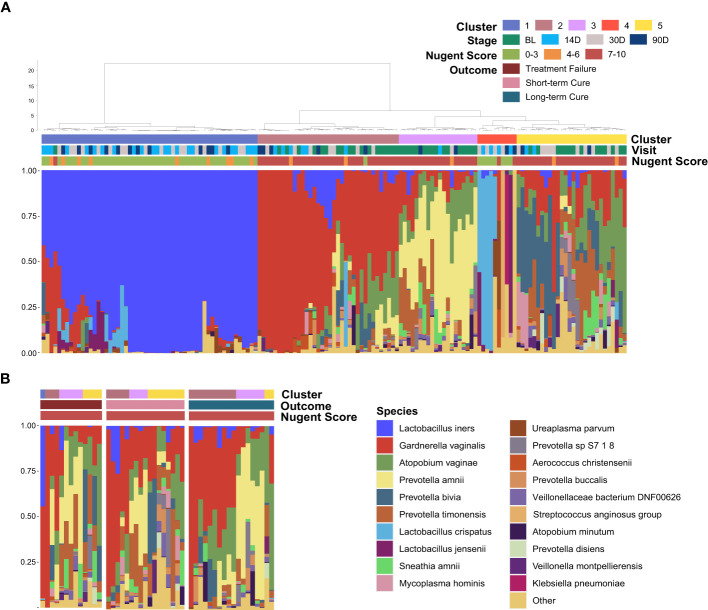
Distribution of the baseline microbiome (BV microbiome) greatly associated with the treatment outcomes. The vaginal microbiome of 59 women sampled longitudinally at all time-points **(A)** and at baseline **(B)**. The samples are hierarchically clustered (R base hclust function with Jensen-Shannon distances and Ward linkage) and classified into five clusters. Nugent Score range, follow-up time-points and treatment outcomes were indicated by the bars.

All of the vaginal samples were grouped into five clusters by hierarchical clustering method (**Figure 3A**). *Lactobacillus iners* was dominant in Cluster 1 (37.16%), and the dominant species in Cluster 4 (6.76%) was varied in samples, including *L. crispatus*, *L. jensenii* and other species with lower average relative abundance. Cluster 2, Cluster 3, and Cluster 5 were highly associated with BV-associated pathogenic bacteria, including *Gardnerella*, *Prevotella*, and *Atopobium* dominated. Among them, a high abundance of *Prevotella amnii* was the main feature of Cluster 3 (12.84%), and Cluster 2 (24.32%) was dominated by *Gardnerella vaginalis*. While *Prevotella bivia*, *Prevotella timonensis*, and *Atopobium vaginae* were the main components of BV-associated pathogens in Cluster 5 (18.92%) and there was no clearly dominant species. It is noteworthy that baseline samples presented more frequently in Cluster 3 (80.00%) than in Cluster 2 (50.00%) or Cluster 5 (50.00%) (P = 0.02&0.03), which demonstrated that distribution regularity could exist in the baseline microbiome (BV microbiome) that may influence the treatment outcomes. To further investigate this issue, we selected the vaginal samples at baseline and re-clustered them according to the outcomes (**Figure 3B**). The result showed that the Cluster composition differed nearly significantly between long-term cure group and short-term cure group (P = 0.059). This suggested a strong relationship between the BV microbiome and long-term cure rate of BV, which could result in the treatment outcomes of *L. gasseri* TM13 and *L. crispatus* LG55.

### 3.4 Differences in vaginal microbiome between the short-term cured subjects and the long-term cured subjects

**Figure 4:**
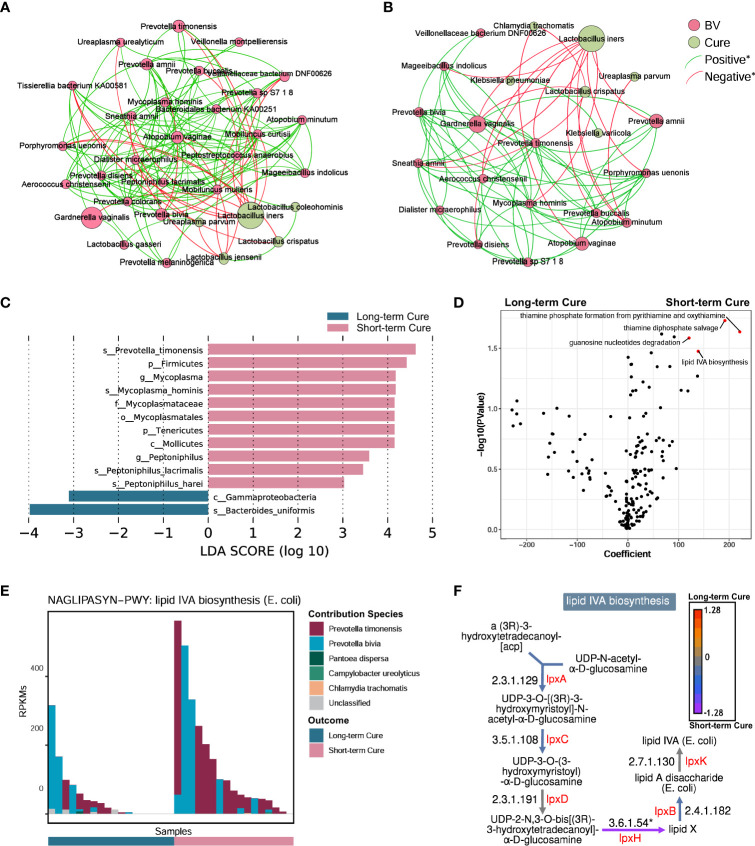
A higher abundance of *Prevotella timonensis* at baseline was significantly associated with long-term cure failure of BV and greatly contributed to the enrichment of the lipid IVA synthesis pathway. Co-occurrence network showing the interbacterial correlations of vaginal microbiome in the short-term cure group **(A)** and long-term cure group **(B)**. Each species is only shown in a color corresponding to the time-point when it has the highest relative abundance. Node size indicates the average abundance of each genus. Lines between nodes represent the interbacterial correlations, and the green line and red line indicate positive and negative correlations, respectively. Histogram of the linear discriminant analysis (LDA) scores computed for species **(C)** with differential abundance between the short-term cure group and long-term group in baseline, and the differential pathways **(D)** found by the linear model (points with p < 0.05 were marked in red). The contribution ranking of the vaginal microbiome to the lipid IVA biosynthesis pathway is shown in the stack bar chart **(E)**. The top of each set of stacked bars indicates the total abundance of the pathway within a single sample. The individual enzymatic steps of the lipid IVA biosynthesis pathway, as well as the differences between the short-term cure group and long-term group of the gene expressing these enzymes, are shown in this flowchart **(F)**. Mann-Whitney U test; *stands for p-value < 0.05.

To describe the differences in core species distribution and their interactions between the short-term cure group (**Figure 4A**) and the long-term cure group (**Figure 4B**), the high-abundance species (mean relative abundance ≥ 0.1%) in vaginal discharge samples at all time-points were selected to perform the co-occurrence network analysis in these two groups. A greater number of high-abundance species of BV-associated pathogenic bacteria and a greater density of positive interspecies interactions was shown in the short-term cure group (106 significant positive correlations) than in long-term cure group (53 significant positive correlations). This suggests that stronger interbacterial interactions help them escape the antibiotic, which is in line with the findings of Gustin’s study (43). *Lactobacillus*, which possessed higher abundance in the cured samples, had the most negative correlations due to the ability of *Lactobacillus* to secrete lactic acid and bactericidal substances, which inhibit the growth of BV-associated pathogenic bacteria (44). In addition, we found *Ureaplasma parvum* enriched in cured samples both in the short-term cured group and in the long-term cured group and a significant negative correlation with certain BV-associated bacteria, which is consistent with the study of Xiao et al. (45). With its small genome (46) and lack of biosynthetic capacity (47), we speculate that *Ureaplasma parvum* inhibits the growth of BV-associated pathogenic bacteria by activating the host immune response.

LEfSe analysis showed differences in vaginal microbiome at baseline between the short-term and long-term cure groups (**Figure 4C**). LDA scores indicated that *Prevotella timonensis*, *Mycoplasma hominis*, *Peptoniphilus lacrimalis*, and *Peptoniphilus harei* were more abundant in the short-term cure; *Bacteroides uniformis* was more abundant in the group with long-term cure. Using the generalized linear model in the MaAsLin2 package, we performed an association analysis of gene copy numbers of functional pathways in the vaginal microbiome at baseline (**Figure 4D**). In the short-term cure group, the gene copy number of lipid IVA synthesis, thiamine phosphate synthesis, and thiamine diphosphate salvage were more abundant than those in the long-term cure group (P < 0.05). Among them, the enrichment of the lipid IVA synthesis pathway in the short-term cure group was mainly caused by the high expression of UDP-2,3-diacylglucosamine diphosphatase encoded by lpxH (**Figure 4F**), and the greatest species contribution was mainly provided by *Prevotella timonensis* (**Figure 4E**), which was consistent with the results of LEfSe analysis.

## 4 Discussion

In this study, there was no significant difference in BV cure rates between the probiotic and control groups at day 14, day 30, and day 90, suggesting a 30-day oral administration of *L. gasseri* TM13 and *L. crispatus* LG55 was ineffective as an adjuvant treatment of BV. The short intervention period may be one of the main reasons for the ineffectiveness compared to other studies that obtained effective intervention results (24, 48). In addition, in the BV conversion population, the percentage of people in a transition state was lower in the probiotic group at all three time-points. This suggests that oral probiotics can modulate and improve vaginal health in cured subjects, which is consistent with previous studies (25, 26). Hence, oral administration of *L. gasseri* TM13 and *L. crispatus* LG55 is effective in restoring the vaginal health of patients recovered from BV.

The inability of the orally administered probiotic strains to reach or colonize the vagina may be the reason of the treatment failure in BV. Similarly, Husain et al. showed that the colonization rate of the intervention strains and the diversity of the vaginal microbiome did not differ between the probiotic and control groups (49). In fact, *L. gasseri TM13* and *L. crispatus LG55* used in this study were isolated, cultured, and screened from the human intestine (33). Existing research has shown that there are still many differences in environmental adaptability and systematic developmental characteristics between *Lactobacillus* strains isolated from the intestine and those isolated from the vagina. For example, *Lactobacillus* isolated from the vagina grows faster in simulated vaginal fluid than those isolated from the intestine. This may be due to inadequate nutrient availability for *Lactobacillus* isolated from the intestine in the vagina. Additionally, *L. crispatus* isolated from the vagina possesses a high abundance of genes related to acid tolerance and carbohydrate-binding modules which help *L. crispatus* better adapt to the acidic environment of the vagina and obtain more nutrients. Differentially, intestine-derived *Lactobacillus* have more genes involved in protecting themselves from viruses or toxins, such as CRISPR/Cas systems, glycoside hydrolases (GHs) family, and tetracycline/lincomycin resistance genes to adapt to the complex intestinal environment (50, 51). Therefore, the difference in ecological niches of strains may be the main reason why intestine-derived *Lactobacillus* cannot colonize in the vagina. A previous multicenter, randomized, double-blind, placebo-controlled trial showed that oral administration of probiotic preparation (prOVag^®^), which contains *Lactobacillus* strains isolated from the vagina, delayed BV recurrence, reduced and vaginal pH and Nugent Score by promoting vaginal *Lactobacillus* counts following standard treatment (52), so *Lactobacillus* strains derived from the vagina may be a better choice for adjunctive treatment of BV. However, whether increased vaginal *Lactobacillus* come from orally administrated probiotics or from the enrichment of vaginal native *Lactobacillus* needs to be further identified. Previous studies have detected probiotics in the vagina using qPCR (48) and 16s rRNA amplicon sequencing (26), but these methods cannot provide strain-specific abundance information, so the conclusions are unreliable. In recent years, strain identification methods based on metagenome sequencing data have emerged, such as MetaMp (53), which uses long-read sequencing technology for strain-level annotation, PStrain (54), based on single nucleotide variants (SNVs), and StrainPanDA (55) that uses pangenome. These advanced methods can provide a more accurate quantitative detection of probiotic colonization and can give direct evidence of the mechanism of action of orally administered probiotics *in vivo*.

In this study, there was no difference in the relative abundance of *L. crispatus* and *L. gasseri* in the vaginal microbiome between probiotic and control groups at all follow-up time-points, which suggest that *L. gasseri* TM13 and *L. crispatus* LG55 mainly influenced the host immune response through the intestine. Oral administration of *L. gasseri* CECT5714 increases the production of SCFAs in the intestine (56) and SCFAs exert anti-inflammatory effects by modulating the levels of PGE, cytokines, and chemokines (57). In addition, consuming fermented milk containing *L. crispatus SMFM2016-NK* can effectively reduce the expression levels of intestinal TNFα and IL-1β (58). Therefore, *L. gasseri* TM13 and *L. crispatus* LG55 may also exert their effects on the improvement of vaginal health by reducing systemic inflammation through immunomodulatory effects in the intestine. In the present study, the abundance of the intervening strains in the intestine could not be maintained at high levels even during the 30-day intervention period. This might be related to the endogenous stability and resilience of the intestinal flora (59), which can prevent the long-term colonization of the intervening strains in the intestine, thus preventing this bacteria from exerting probiotic functions in a sustained and stable manner. Our results also support this inference; the largest difference between the probiotic and control groups in the proportion of participants with Nugent Score < 4 occurred on day 14. Therefore, to improve the effect of *L. gasseri TM13* and *L. crispatus LG55*, a longer intervention period might be one of the optimization options.

We found that *L. iners* had the highest average relative abundance in cured patients’ vaginal samples. Compared with other *Lactobacillus* spp., *L. iners* has lower D-lactic acid production, more complex nutritional structure and more variable Gram staining morphology (60). In addition, *L. iners* can encode a cytotoxin, which is a pore-forming toxin with a similar structure to the vaginolysin encoded by *Garderella vaginalis *(61). This may be the reason why *L. iners* can disturb the balance of vaginal microbiota (62). However, a recent study found that BV patients with more *L. iners* in their baseline vaginal microbiota had a better clinical treatment outcome after receiving 5% MET gel for 5 days, indicating limited antibacterial function of *L. iners* may play a key role in the treatment of BV (63). In summary, it is necessary to further clarify whether *L. iners* is a friend or foe to vaginal health in the future.

The abundance of key bacteria in the vagina influences whether BV can be cured in the long term (45, 64). We found that *Prevotella timonensis* is enriched in the vagina at the baseline in long-term cure failure patients and that the lipid IVA synthesis pathway, which is abundantly enriched in *Prevotella timonensis*, is an important precursor material for LPS biosynthesis. *Prevotella timonensis* is a strictly anaerobic Gram-negative bacterium enriched in the genital tract of BV (65), *Chlamydia trachomatis* infection (66), and HPV infection (67).Nienke et al. showed that *Prevotella timonensis* induces the maturation of DC cells to secrete large amounts of pro-inflammatory cytokines, including IL-1β and IL-8, to enhance the inflammatory response in the genital tract (68). Our results also showed a significantly positive correlation between *Prevotella timonensis* and Lipid IVA synthesis. Lipid IVA is the lipopolysaccharide (LPS) A tetra-acetylated precursor of lipid A in biosynthesis. Although Lipid IVA has been reported as a structural antagonist of LPS, it could also be a substrate of LPS synthesis and further is able to activate the innate immune responses via TLR4 and its co-receptor MD-2 (69). We speculate that *Prevotella timonensis* aggravated the inflammatory effect by producing LPS persistently, which would induce long-term cure failure of BV. But the pro-inflammatory mechanism of *Prevotella timonensis* in the vagina and how it perturbed the vaginal microbiome and host immune response still need further study. Furthermore, the probiotic effect of oral *L. gasseri TM13* and *L. crispatus LG55* on the genital tract has a limited effect in curing subjects, suggesting that precise treatment and personalized probiotic interventions are necessary for BV patients.

This study has some limitations. In terms of the clinical trial design, the participants completed their trials when diagnosed with BV, and there was no continuous follow-up for these patients. We were unable to provide a comprehensive description of the microbiome dynamics of BV treatment failure. Regarding bioinformatics analysis techniques, the relative abundance at the species level was insufficient to accurately calculate the number of intervention probiotic strains in the samples. In addition, the relatively small sample size is also one of the limitations of this study. Finally, future microbiome studies on probiotic interventions to assist in treating BV need to be supported by larger sample sizes and strain-level annotation techniques with higher resolution.

## 5 Conclusions

Although orally administrated *L. gasseri* TM13 and *L. crispatus* LG55 cannot improve BV cure rates, it restores vaginal health after cure mainly acting through the intestine. A higher abundance of *Prevotella timonensis* at baseline was significantly associated with long-term cure failure of BV and greatly contributed to the enrichment of the lipid IVA synthesis pathway, which could aggravate inflammation response. This inferred that individualized intervention mode should be developed to restore the vaginal health of patients recovering from BV.

## Data availability statement

The data presented in the study are deposited in the Genome Sequence Archive (70) in National Genomics Data Center (71), accession number HRA004429, and the CNGB Sequence Archive (72) of China GeneBank DataBase (73) with accession number CNP0003852. Please refer them at https://ngdc.cncb.ac.cn/search/?dbId=&q=HRA004429 and https://db.cngb.org/search/project/CNP0001543.

## Ethics statement

The studies involving human participants were reviewed and approved by Medical Ethnic Committee of Peking University Shenzhen Hospital. The patients/participants provided their written informed consent to participate in this study.

## Author contributions

XZ, LX, and LG designed the research. SF, JL, LH, XL, YL, and YZh carried out the clinical trial. XZ, LG, FQ, JL and SZ supported the clinical trial and organized data. FQ, CF, ZH, YZo and SZ analyzed the data. FQ, XZ, CF, and ZH draft the paper. LX, SF and LG provided critical revisions of the article. LG, YZh, and HZ were responsible for the probiotic product support. XZ and XL supervised the project. All authors contributed to the article and approved the submitted version.

## Supplementary material

Supplementary Figure 1 Shannon and Simpson index between the probiotics group and the control group in all time-points **(A)**. PCoA-based Bray-Curtis distance matrices between the probiotics group and the control group in all time-points **(B)**.
