# Supplementary figures and images for: Orally administrated Lactobacillus gasseri TM13 and Lactobacillus crispatus LG55 can restore the vaginal health of patients recovering from bacterial vaginosis
Source: Front Immunol. 2023 Jul 27;14:1125239. doi: 10.3389/fimmu.2023.1125239 (PMC10415204; doi:10.3389/fimmu.2023.1125239)

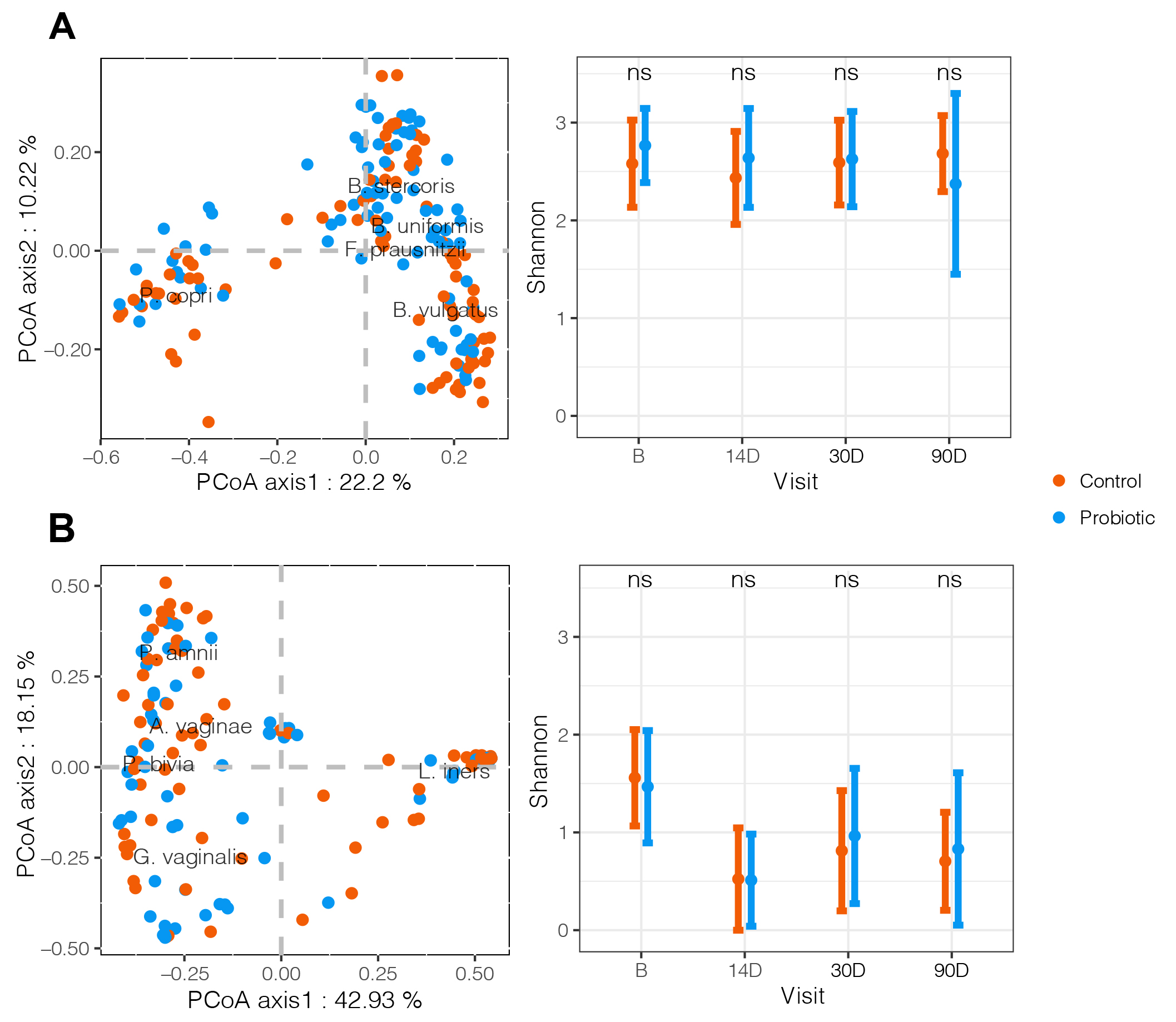

Supplement: Supplementary Figure 1 — Shannon and Simpson index between the probiotics group and the control group in all time-points (A). PCoA-based Bray-Curtis distance matrices between the probiotics group and the control group in all time-points (B). [file Image_1.jpeg]
